# Zinc- and bicarbonate-dependent ZIP8 transporter mediates selenite uptake

**DOI:** 10.18632/oncotarget.9205

**Published:** 2016-05-06

**Authors:** Joseph R. McDermott, Xiangrong Geng, Lan Jiang, Marina Gálvez-Peralta, Fei Chen, Daniel W. Nebert, Zijuan Liu

**Affiliations:** ^1^ Department of Biological Sciences, Oakland University, Rochester, MI 48309, USA; ^2^ Department of Environmental Health and Center for Environmental Genetics, University of Cincinnati Medical Center, Cincinnati, OH 45267, USA; ^3^ Department of Pharmaceutical Sciences, College of Pharmacy and Health Sciences, Wayne State University, Detroit, MI 48201, USA

**Keywords:** selenite, zinc, micronutrient, ZIP8, membrane-bound transporter

## Abstract

Selenite (HSeO_3_^−^) is a monovalent anion of the essential trace element and micronutrient selenium (Se). In therapeutic concentrations, HSeO_3_^−^ has been studied for treating certain cancers, serious inflammatory disorders, and septic shock. Little is known, however, about HSeO_3_^−^ uptake into mammalian cells; until now, no mammalian HSeO_3_^−^ uptake transporter has been identified. The ubiquitous mammalian ZIP8 divalent cation transporter (encoded by the *SLC39A8* gene) is bicarbonate-dependent, moving endogenous substrates (Zn^2+^, Mn^2+^, Fe^2+^ or Co^2+^) and nonessential metals such as Cd^2+^ into the cell. Herein we studied HSeO_3_^−^ uptake in: human and mouse cell cultures, shRNA-knockdown experiments, *Xenopus* oocytes, wild-type mice and two transgenic mouse lines having genetically altered ZIP8 expression, and mouse erythrocytes *ex vivo*. In mammalian cell culture, excess Zn^2+^ levels and/or ZIP8 over-expression can be associated with diminished viability in selenite-treated cells. Intraperitoneal HSeO_3_^−^ causes the largest ZIP8-dependent increases in intracellular Se content in liver, followed by kidney, heart, lung and spleen. In every model system studied, HSeO_3_^−^ uptake is tightly associated with ZIP8 protein levels and sufficient Zn^2+^ and HCO_3_^−^ concentrations, suggesting that the ZIP8-mediated electroneutral complex transported contains three ions: Zn^2+^/(HCO_3_^−^)(HSeO_3_^−^). Transporters having three different ions in their transport complex are not without precedent. Although there might be other HSeO_3_^−^ influx transporters as yet undiscovered, data herein suggest that mammalian ZIP8 plays a major role in HSeO_3_^−^ uptake.

## INTRODUCTION

Selenite (HSeO_3_^−^) is an ion of the essential trace metal and micronutrient selenium (Se). Using therapeutic doses, selenite has been studied for treatment of cancer, serious forms of inflammation, septic shock [1], and other conditions such as heavy-metal detoxication, radioprotection, and wound-healing [2–10]. Whereas physiological serum Se levels are 80−120 μg/L (~1.3 μM), serum Se levels after therapeutic HSeO_3_^−^ treatment range between 1.5 μM and 20 μM. High doses of HSeO_3_^−^ can adversely affect signal-transduction pathways and induce oxidative stress [11–13].

Selenite reacts spontaneously with protein thiols, *e.g.* neighboring cysteines in thioredoxin folds, and the two or four cysteines coordinated in zinc-finger proteins [14, 15]. Selenite is unusual in its capacity to oxidize such targets–normally redox-protected–without cellular reduced glutathione (GSH) first being depleted [16, 17].

Numerous important cell-signaling and apoptosis pathways are regulated by selenite—including NF-κB, IKK, PKC-δ, androgen receptor, the AKT-activated p38MAPK-eIF4E axis [10, 18–20]. Although selenite involvment has been identified in these pathways, it has not yet been established with certainty how selenite enters the cell, or how intracellular Se levels are regulated.

Because of its instability, HSeO_3_^−^ reactions do not appear to be regulated enzymatically. We therefore hypothesized that membrane transport might be pivotal in controlling intracellular Se levels. However, until now no mammalian selenite transporter has been identified.

Certain human cancer cell lines, when treated with selenite, display greater Se accumulation than normal cells, an effect purported to be associated with preferential selenite-induced toxicity in malignant cells [21–23]. Because the prostate cancer cell line DU145 is known to be hypersensitive to selenite [11, 24–26], we postulated that selenite uptake might be particularly robust in this cell line. DU145 cells were therefore used to screen various small molecules and ions for effects on selenite uptake; these initial studies indicated that zinc (Zn^2+^) was the best candidate for stimulating intracellular Se accumulation following HSeO_3_^−^ treatment.

Searching the literature for transporters that move Zn^2+^ into the cell, we became aware of mammalian ZIP8—a divalent cation- and bicarbonate-dependent transporter (encoded by the *SLC39A8* gene) that imports the essential metals Zn^2+^, Mn^2+^, Fe^2+^ and Co^2+^, as well as the nonessentical metal Cd^2+^ [27–31]. The present study, using numerous heterologous expression systems and transgenic mice, provides evidence strongly supporting a model of zinc- and bicarbonate-dependent ZIP8-mediated selenite uptake.

## RESULTS

### Dependence of selenite uptake on Zn^2+^ and ZIP8

**Figure 1:**
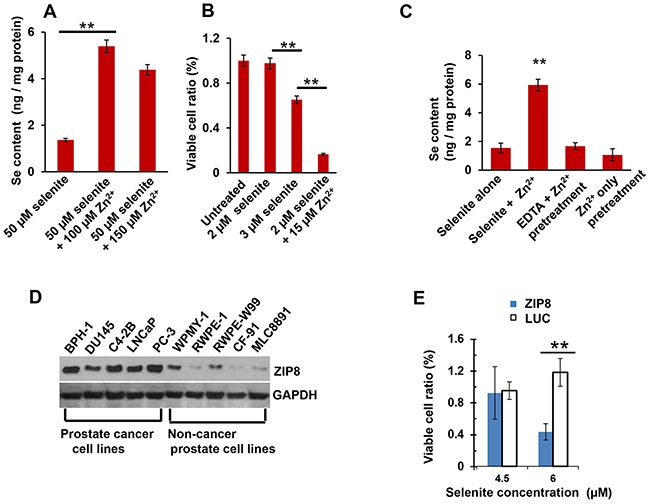
Intracellular Se content and toxicity; ZIP8 protein levels in human prostate cancer cells **A.** effect of Zn^2+^ on Se content in selenite-treated DU145 cells; Zn^2+^ added concomitantly with HSeO_3_^−^ for 30 min. **B.** effect of Zn^2+^ on selenite-mediated viability of DU145 cells following 12-h treatment, varying HSeO_3_^−^ amounts. Untreated cultures were given a ratio value of **1.0**., and other ratios of experimental regimens are expressed, relative to **1.0**. **C.** effect of EDTA and Zn^2+^ on Se content in selenite-treated cells; EDTA (5 mM) + Zn^2+^ (200 μM) pretreatment was 5 min; at *far right*, Zn^2+^ was washed out prior to HSeO_3_^−^ treatment for 30 min. **D.** Western blots of human ZIP8 in five prostate cancer lines (*left lanes*) and five prostate non-cancer cell lines (*right lanes*). GAPDH, glyceraldehyde 3-phosphate dehydrogenase, lane-loading control. **E.** cell viability of Zn^2+^-treated ZIP8-MEFs versus LUC-MEFs, as a function of selenite concentration; treatment (5 μM Zn^2+^ and 25 mM HCO_3_^−^) was 16 h. For panels B and **E,** “relative viability” of cells not treated with selenite is given a value of 1.0, and viability of all other experimental regimens are expressed relative to that control.

When human prostate cancer DU145 cells were treated with HSeO_3_^−^ for 30 min, levels of intracellular Se content were detectable; Zn^2+^ treatment increased Se content ~4-fold (Figure 1A, *1st & 2nd bars from left*). Further increases in Zn^2+^ concentration did not augment Se accumulation, likely because the co-transport substrate had reached saturation. Slightly (50%) higher concentrations of Zn^2+^ diminished Se content; this might reflect a slight toxic effect but was not statistically significant (Figure 1A, *compare 2nd & 3rd bars*).

During 12-h treatments, whereas 2 μM HSeO_3_^−^ showed no detectable toxicity, statistically significant loss of cell viability was seen with 3 μM HSeO_3_^−^ (Figure 1B, *compare 1st, 2nd & 3rd bars*). Zn^2+^ treatment of 2 μM selenite-treated DU145 cells caused ~85% loss of viability (Figure 1B, *compare 2nd & 4th bars*). Hence, at sufficiently high doses, Zn^2+^ treatment is able to potentiate selenite-induced cytotoxicity ~6-fold.

The ~4-fold increase in Se content with HSeO_3_^−^ + Zn^2+^, compared with HSeO_3_^−^ alone, is illustrated again in Figure 1C (*1st two bars*). When extracellular Zn^2+^ was removed by EDTA chelation during preincubation (Figure 1C, *compare 2nd & 3rd bars*), zinc-mediated increases in intracellular Se content were not seen.

As described earlier, our initial screen in DU145 cells had demonstrated that Zn^2+^ was most successful at increasing intracellular Se accumulation, as well as enhancing selenite-associated toxicity; hence, we examined ZIP8 protein levels (Figure 1D). ZIP8 protein was found to be highly expressed in DU145 and four other human prostate cancer cell lines, whereas five non-cancer prostate lines displayed little, or negligible, amounts of ZIP8 protein.

Consistent with the preceding results, we also found that viability in selenite-treated cells is affected by ZIP8 expression levels (Figure 1E). Selenite-treated (high-ZIP8-expressing) ZIP8-MEFs showed more toxicity than selenite-treated (low-ZIP8-expressing) LUC-MEF control cells.

### Zinc- and bicarbonate-dependent selenite uptake by ZIP8 in *Xenopus* oocytes and mouse cell cultures

**Figure 2:**
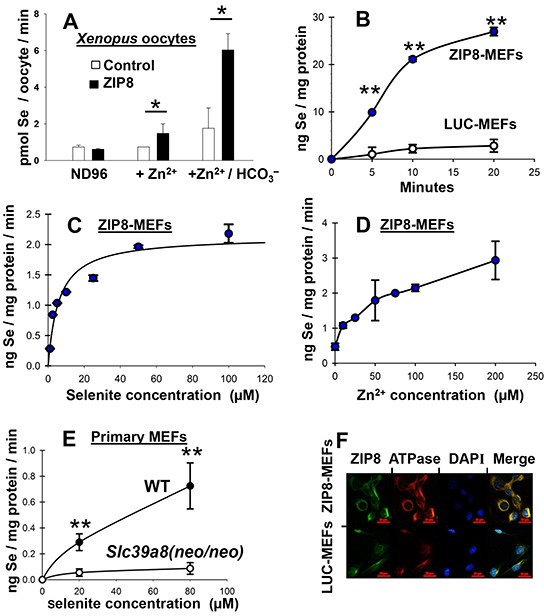
Se uptake as a function of time and Zn^2+^ concentration in oocytes and mouse cell cultures; localization of ZIP8 to plasma membrane **A.** Se content following addition of Zn^2+^ (500 μM), or Zn^2+^ + HCO_3_^−^ (3.5 mM) in selenite-treated *Xenopus laevis* oocytes expressing mouse ZIP8 cDNA (*closed bar*) versus control oocytes carrying vector only (*open bar*). Without added HCO_3_^−^, note that a basal level of HCO_3_^−^ exists in the ND96 medium due to pCO_2_ in solution, as can be calculated by the Henderson-Hasselbach equation [30]. **B.** Se uptake kinetics in stably-transfected high-ZIP8-expressing ZIP8-MEFs vs control low-ZIP8-expressing LUC-MEFs during 20-min exposure to 50 μM HSeO_3_^−^ + 100 μM Zn^2+^. **C.** Intracellular Se content in ZIP8-MEFs as a function of HSeO_3_^−^ concentration; cells were treated with Zn^2+^ (100 μM) plus 5 to 100 μM HSeO_3_^−^. **D.** Intracellular Se content in ZIP8-MEFs (treated with 50 μM HSeO_3_^−^ for 20 min) as a function of Zn^2+^ concentration. **E.** Intracellular Se content as a function of HSeO_3_^−^ concentration (20-min treatment) in wild-type (WT) vs *Slc39a8(neo/neo)* knockdown mouse primary embryonic fibroblast (MEF) cultures; Zn^2+^ (100 μM) and HCO_3_^−^ (25 mM) were constant. **F.** confocal microscopy of co-localization of ZIP8 (*green*) with the membrane marker Na^+^/K^+^-ATPase subunit (*red*) in ZIP8-MEF versus LUC-MEF cultures. DAPI, *blue* stain for DNA, *i.e.* nucleus. For all mouse culture experiments, HCO_3_^−^ was present in culture medium at 25 mM. Addition of the HCO_3_^−^ concentrations used in these experiments in either frog oocytes or mouse cultures did not significantly alter pH (7.5) of the medium. *Brackets* denote S.D. *P <0.05, **P <0.01.

The *Xenopus laevis* oocyte model is a favorite for mammalian transporter research because *Xenopus* oocytes contain few endogenous transporters; moreover, the oocytes exhibit negligible passive HSeO_3_^−^ uptake. We found that ZIP8 expression in selenite-treated oocytes is associated with increased Se content, and Se content is greatly enhanced by addition of HCO_3_^−^ (Figure 2A). ZIP8-mediated Zn^2+^ uptake had previously been demonstrated to require HCO_3_^−^ in *Xenopus* oocytes [30], as well as mouse cell cultures [28].

In stably-transfected ZIP8-expressing mouse embryonic fibroblasts (ZIP8-MEFs) compared with luciferase-expressing control MEFs (LUC-MEFs), the rate of intracellular Se accumulation after HSeO_3_^−^ treatment for 20 min was 8-fold higher in ZIP8-MEFS than in LUC-MEFs (Figure 2B). Note that Se content in selenite-treated LUC-MEFs does indeed increase slightly with time (Figure 2B); this is consistent with existence of endogenously-expressed ZIP8 even in LUC-MEFs, whereas the higher Se accumulation in ZIP8-MEFs reflects ZIP8 over-expression due to the ZIP8 cDNA transfected via the pRevTre vector (in addition to the endogenously-expressed ZIP8). These MEF cell lines had previously been used to characterize ZIP8-mediated divalent cation transport [28].

In selenite-treated ZIP8-MEF uptake studies (Figure 2C), the apparent Km of HSeO_3_^−^ transport was estimated at 5.9 μM; this Km-value is within the range of HSeO_3_^−^ concentrations used in lab animal and cell culture studies, as well as in clinical trials, but appears to be higher than that reflected by minimal daily dose requirements as a micronutrient. In ZIP8-MEFs (Figure 2D), intracellular Se content was increased by ~4-fold at 50 μM Zn^2+^, and by ~6-fold at 200 μM Zn^2+^.

In selenite-treated wild-type (WT) primary MEF cultures (Figure 2E), Se content was ~9-fold greater than that in *Slc39a8(neo/neo)* MEFs with 80 μM HSeO_3_^−^. As mentioned above, *Slc39a8* knockdown mice exhibit substantially lower ZIP8 mRNA and protein levels than WT mice [32]. The Figure 2E data show that genetically-engineered ZIP8 levels in selenite-treated primary embryo cell cultures of two different types of mice are also associated intracellular Se content—similar to that observed in stably transformed ZIP8-MEFs (Figure 2B, 2C & 2D) and *Xenopus* oocytes (Figure 2A).

ZIP8 expression was observed by immunofluorescence in permeabilized cells (Figure 2F), or without permeabilization (*data not shown*), revealing substantial amounts of ZIP8 protein on the cell surface, as expected. Membrane location was confirmed by co-localization with a membrane marker, the Na^+^/K^+^-ATPase subunit-1β, the Na^+^/K^+^-ATPase subunit-1 (Figure 2F), consistent with the previous detailed studies in mouse stably-transfected cell cultures [28].

### ZIP8 down-regulation is associated with decreased selenite and zinc uptake

**Figure 3:**
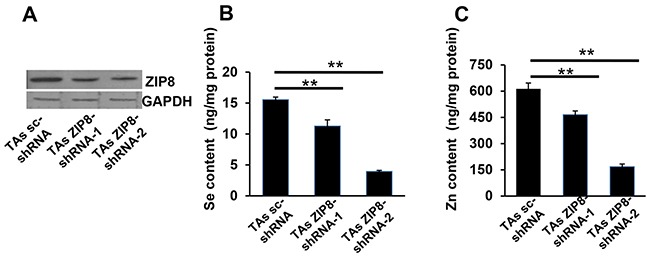
ZIP8 knockdown by shRNA causes decreased ZIP8 protein expression and Se and Zn uptake **A.** Western blot of human ZIP8 in TAs cells stably expressing shRNA against ZIP8 mRNA (shRNA-1 and shRNA-2) vs scrambled shRNA (sc-shRNA) control; GAPDH, glyceraldehyde 3-phosphate dehydrogenase, lane-loading control. **B.** Se content and **C.** Zn content in TAs cells, ZIP8-knockdown vs control, after exposure to HSeO_3_^−^ + Zn^2+^ (both 200 μM) for 30 min. TAs_ZIP8-shRNA-1 and TAs_ZIP8-shRNA-2 represent two different stably-expressing cell lines carrying two different shRNAs. For all experiments, HCO_3_^−^ (25 mM) was present in the culture medium. Intracellular Se and Zn content was determined by ICP-MS. *Brackets* denote S.E.M. *P <0.05, **P <0.01.

To study HSeO_3_^−^ and Zn^2+^ uptake in ZIP8-knockdown cells, we first created DU145 cell lines stably expressing shRNAs targeted against ZIP8 mRNA (ZIP8-shRNA). Despite successful expression of stably-transfected ZIP8-targeted shRNAs, however, significant decreases in ZIP8 levels were not found in DU145 cells (data not shown). Next, we chose TAs cells (Chang *et al.*, 2014), and this cell line was successful for shRNA-mediated ZIP8-knockdown studies. Comparing control with two different stably-expressing ZIP8 knockdown lines carrying two different shRNAs targeted against ZIP8 mRNA, we found ZIP8 protein amounts onWestern blots to be <50% lowered by both ZIP8-shRNAs (Figure 3A). Treatment of these TAs cells with both HSeO_3_^−^ and Zn^2+^ together resulted in decreases in intracellular Se content (Figure 3B) and intracellular Zn^2+^ content (Figure 3C), in proportion to the shRNA-mediated ZIP8 knockdown efficiency. Again, these data are consistent with a specific association between ZIP8 protein levels, and both lowered HSeO_3_^−^ and Zn^2+^ uptake, and less intracellular Se and Zn accumulation.

### Correlation of tissue Se content with ZIP8 levels in genetically different selenite-treated mice

**Figure 4:**
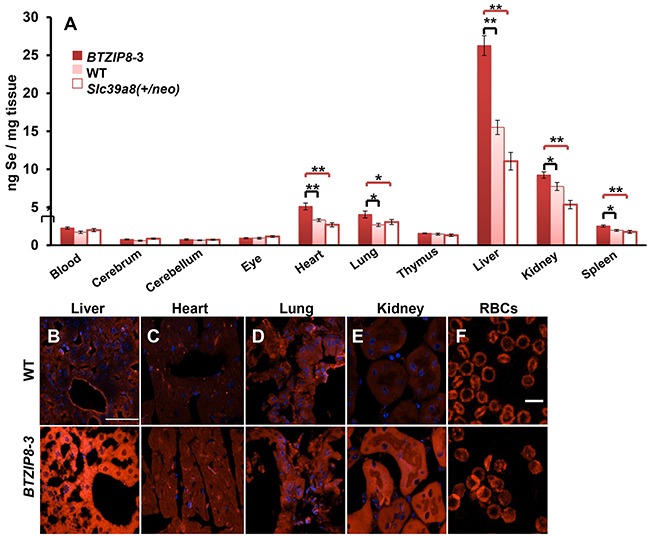
Se content in ten mouse tissues as a function of ZIP8 concentrations in three mouse lines having different *Slc39a8* genotypes **A.** Intracellular Se content, 2 h after IP selenite (2.5 mg/kg) administration to the ZIP8-over-expressing *BTZIP8-*3 mouse line (*solid red bar*; N=6), the ZIP8-normal-expressing wild-type (WT; *pink bar*; N=9), and the hypomorph ZIP8 *Slc39a8(+/neo)* line; (*open bar*; N=9). *Red brackets* (S.E.M.) depict statistical differences between *BTZIP8*-3 and *Slc39a8(+/neo)*; *black brackets* (S.E.M.) signify statistical differences between *BTZIP8-3* and WT. *P <0.05, **P <0.01. Animals (2-3 months of age) included similar numbers of males and females; we first had determined that no sex differences existed. **B–F.** ZIP8 immunofluorescence in 10-μm tissue sections from WT vs *BTZIP8-3* mice. *White bar* (in *panel B*) = 50 μm for *panels B through E*; *white bar* (in *panel F*) = 10 μm.

Whereas wild-type (WT) mice have the usual (diploid) two copies of the functional *Slc39a8* gene, *BTZIP8-3* mice carry five *Slc39a8* gene copies, resulting in ~2.5-fold higher expression of ZIP8 mRNA and protein in all tissues examined [33]. On the other hand, *Slc39a8(neo/neo)* mice are hypomorphic, and die by 24 h postpartum; therefore, for experiments using adult mice (Figure 4), we used “knockdown” *Slc39a8(+/neo)* heterozygotes—in which ZIP8 expression is substantially less than that of WT mice—but which are still viable and fertile [32]. It should be noted that sufficient concentrations of Zn^2+^ and HCO_3_^−^ always exist in tissues of the intact animal; *i.e.* there was no need to add exogenous Zn^2+^ or HCO_3_^−^.

Se content in control tissues of the untreated three mouse genotypes was negligible, *i.e.* Se background level in liver averaged ~0.2 ng/mg tissue, which was <10% of any Se concentration detected of any of the ten tissues in any of the three selenite-treated mouse genotypes (*data not shown*). Following HSeO_3_^−^ treatment, substantial genetic differences in Se content were found in heart, lung, liver, kidney and spleen; on the other hand, low Se content, and no statistically significant differences among the three genotypes, were seen in whole blood, cerebrum, cerebellum, eye or thymus (Figure 4A). Interestingly, although kidney and lung are known to have the highest ZIP8 levels [27], the highest Se content was observed in liver—*BTZIP8-3* revealed ~2.3-fold greater Se content than *Slc39a8(+/neo)* mice; this observation might reflect the essential role of liver in Se metabolism and storage.

In each tissue having statistically significant Se levels, the three mouse genotypes [*BTZIP8-3*, WT, and *Slc39a8(+/neo)*] roughly paralleled the genetically different ZIP8 levels (Figure 4A). Comparison of ZIP8 protein concentrations in liver, heart, lung, kidney, and RBCs (Figure 4, *panels* 4B–4F), confirmed that *BTZIP8-3* mice, carrying five *Slc39a8* copies [33], express greater amounts of ZIP8 than WT mice, which harbor the normal diploid copy number of two *Slc39a8* genes.

### Plasma zinc is sufficient for ZIP8-mediated selenite transport in RBCs

We found that mouse erythrocytes (red blood cells; RBCs) have substantial ZIP8 expression; this provided a convenient *ex vivo* assay to study the role of endogenous Zn^2+^ in ZIP8-mediated HSeO_3_^−^ transport, which is easier than assays in *Xenopus* oocytes or mammalian cell cultures.

**Figure 5:**
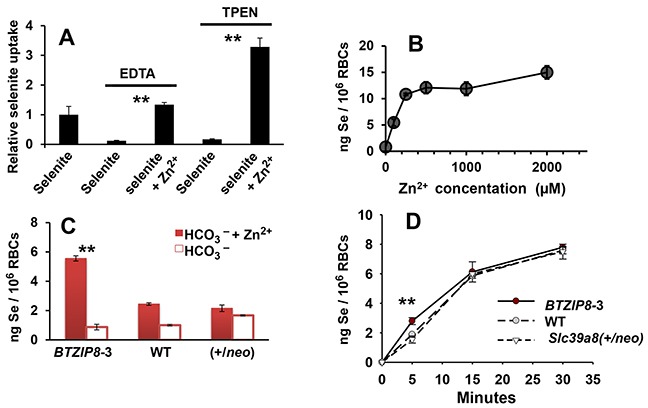
Se content in mouse whole blood or washed red blood cells (RBCs) **A.** relative Se content in WT whole blood, following HSeO_3_^−^ treatment (20 μM; 15 min at 37°C), with, versus without, Zn^2+^ (75 μM); where indicated, whole blood was pretreated for 5 min with EDTA (4 mM) or TPEN (100 μM). Value for selenite-treated whole blood without added Zn^2+^ was chosen as “**1.0**” and all other values are relative to that. **B.** Intracellular Se content in isolated WT RBCs resuspended in PBS containing HCO_3_^−^ (10 mM; 15 min at 37°C), as a function of Zn^2+^ concentration following 5-min pretreatment with HSeO_3_^−^ (20 μM, 37°C). **C.** Intracellular Se content in isolated RBCs from the three mouse genotypes resuspended in PBS with HCO_3_^−^ (25 mM) + 75 μM Zn^2+^ (*closed red bars*) versus 25 mM HCO_3_^−^ alone (*open bars*). Cells were incubated with HSeO_3_^−^ (20 μM) for 5 min at 37°C. **P <0.01, comparing *BTZIP8-3* with, vs without, added Zn^2+^. **D.** comparison of rate of Se uptake in RBCs (also resuspended in PBS), as a function of incubation time (37°C); RBCs were isolated from *BTZIP8-3*, WT and *Slc39a8(+/neo)* mice; RBCs were treated with HSeO_3_^−^ (20 μM). **P <0.01, comparing *BTZIP8-3* with both WT and *Slc39a8(+/neo)*. Brackets denote S.E.M.

After incubating whole blood from WT mice for 15 min with 20 μM HSeO_3_^−^ (Figure 4A), we chose the amount of intracellular Se content as “standard value of 1.0”; all other treatments are expressed relative to this value. When Zn^2+^ in mouse plasma was removed by EDTA chelation, Se content was diminished ~10-fold (Figure 5A, *compare 1st & 2nd bars*). Preincubation with another zinc chelator TPEN produced similar lowering of intracellular Se content (Figure 5A, *compare 1st & 4th bars*). To verify that these striking decreases in Se content represent Zn^2+^ removal, we incubated RBCs with 75 μM Zn^2+^ for 20 min subsequent to the EDTA or TPEN chelation pretreatment; Se content was restored to levels even higher than basal levels (Figure 5A, *compare 1st, 3rd & 5th bars*). These data likely reflect the fact that the Zn^2+^ level (75 μM), added exogenously after chelation, is higher than physiological plasma Zn^2+^ levels.

Following HSeO_3_^−^ incubation of washed RBCs from WT mice for 15 min, Se content in RBCs was maximal at 150-250 μM Zn^2+^, with little increase even at 2.0 mM Zn^2+^ (Figure 5B). Based on TPEN concentrations required for complete inhibition of Se uptake, we estimated the amount of available endogenous plasma Zn^2+^ is <25 μM. Incubating washed RBCs from the three mouse genotypes with HSeO_3_^−^ and 25 mM HCO_3_^−^ for 15 min (Figure 5C) resulted in measurable Se uptake, but Se content was increased when 75 μM Zn^2+^ was also added—with the over-expressing ZIP8 *BTZIP8-3* showing a much larger zinc response than the diploid WT or the *Slc39a8(+/neo)* knockdown mouse RBCs.

Interestingly, looking at Se content of RBCs as a function of time (Figure 5D), we found the difference between the *BTZIP8-3* RBCs, and both the WT and *Slc39a8(+/neo)* RBCs, was statistically significant after 5 min incubation, but this was no longer observed after 15 or 30 min incubation. These results likely reflect the maximal ZIP8 capability and efficiency (saturation) for HSeO_3_^−^ uptake in *BTZIP8-3* RBCs, in which, with longer incubation times, the lower-ZIP8-expressing levels in WT and *Slc39a8(+/neo)* RBCs become equalized.

### Independence of ZIP8-mediated selenite uptake from the extracellular thiol-conversion pathway

Lastly, we wished to compare the contribution of HSeO_3_^−^ uptake via the ZIP8 transporter versus the contribution of HSeO_3_^−^ uptake via the proposed extracellular thiol-conversion model. GSH is a commonly used potent selenite-reducing thiol. EDTA (5 mM) did not prevent HSeO_3_^−^ reduction to Se° in water by excessive amounts (100 μM; or even 800 μM) of GSH; in fact, EDTA slightly increased the formation of Se^0^ (*data not shown*).

**Figure 6:**
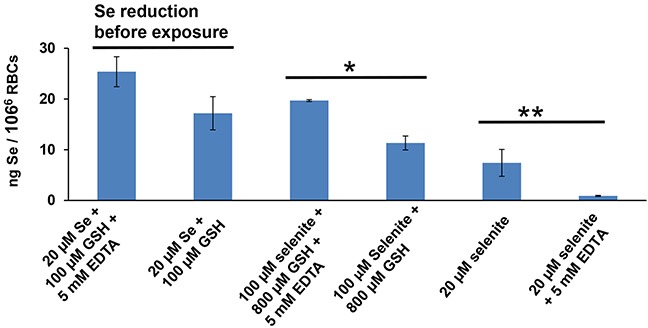
Intracellular Se content of RBCs (resuspended in PBS, without any exogenous Zn^2+^ or HCO_3_^−^), in response to thiol, HSeO3−, and/or EDTA RBCs were incubated for 2 h with HSeO_3_^−^ or its reduced products (20 or 100 μM): *first two bars* denote first being reduced for 15 min by GSH (100 μM) alone, causing build-up of extracellular reduced selenium product(s) (derived from 20 μM HSeO_3_^−^, or with GSH plus EDTA (5 mM); *second two bars* indicate no reduction by GSH first, but higher concentrations of HSeO_3_^−^ treatment (100 μM), comparing GSH (800 μM) with or without 5-min EDTA pretreatment (5 mM); *last two bars* designate HSeO_3_^−^ treatment (20 μM) alone or with 5-min EDTA (5 mM) pretreatment, but without GSH. *P < 0.05, **P < 0.01.

Following reduction of 20 μM HSeO_3_^−^ by GSH for 15 min, with or without EDTA (Figure 6, *1st two bars*), RBCs were incubated for 2 h—with the resulting reaction mixture (containing Se^0^, HSe^−^; perhaps other reduced products); Se content was slightly higher with EDTA, but did not reach statistically significant levels (*P* = 0.053). Thus, comparing the ZIP8-mediated HSeO_3_^−^ transport and thiol-conversion pathway, EDTA markedly decreased ZIP8-mediated HSeO_3_^−^ uptake, whereas EDTA had an opposite effect—perhaps even a slight stimulatory effect—on the extracellular thiol-conversion pathway.

When RBCs were incubated with even larger concentrations of HSeO_3_^−^ and GSH, without prior reduction *in vitro* (Figure 6, *3rd & 4th bars*), EDTA preincubation significantly increased Se content (*P* <0.05), compared to RBCs without EDTA preincubation. Therefore, increased Se content does not occur when reactants are added directly to the cells without prior reduction *in vitro*. And, without GSH in the system (Figure 6, *5th & 6th bars*), EDTA pretreatment dramatically lowered Se content when comparing EDTA-pretreated with no EDTA pretreatment of selenite-treated RBCs.

These observations strongly suggest that prevention by EDTA of HSeO_3_^−^ uptake—when HSeO_3_^−^ and exogenous Zn^2+^ are added to cells in culture (Figure 1C), or when HSeO_3_^−^ is added to RBCs having sufficient endogenous Zn^2+^ (Figure 6), is not a consequence of inhibiting thiol-mediated uptake. Thus, we conclude that contribution of any endogenous extracellular thiol-conversion pathway to these mammalian HSeO_3_^−^ uptake systems is negligible.

## DISCUSSION

Herein we have shown—without exception—that the level of membrane-bound ZIP8 divalent cation/bicarbonate transporter protein is associated with the amount and rate of selenite (HSeO_3_^−^) uptake and, hence, intracellular selenium (Se) content. We have demonstrated this by using several complementary heterologous experimental models: human prostate cancer vs normal prostate cells in culture, *Xenopus* oocytes *ex vivo*, stably-expressing shRNA-targeted ZIP8 knockdown cells, stably-transfected high-ZIP8-expressing mouse embryo fibroblasts (ZIP8-MEFs) versus low-ZIP8-expressing LUC-MEFs, wild-type (WT) red blood cells (RBCs) *ex vivo*, and WT mice, as well as the ZIP8-overexpressing mouse (*BTZIP8-3*) and the ZIP8 hypomorphic knock-down *Slc39a8(+/neo)* mouse. Moreover, in each model system, HSeO_3_^−^ uptake is consistently dependent on the presence of sufficient amounts of Zn^2+^ and HCO_3_^−^, which parallels the previously established zinc- and bicarbonate-dependent properties of ZIP8 function. Together, these data strongly implicate that the ZIP8 importer protein plays a major role in HSeO_3_^−^ uptake and, hence, regulation of intracellular Se concentrations.

**Figure 7:**
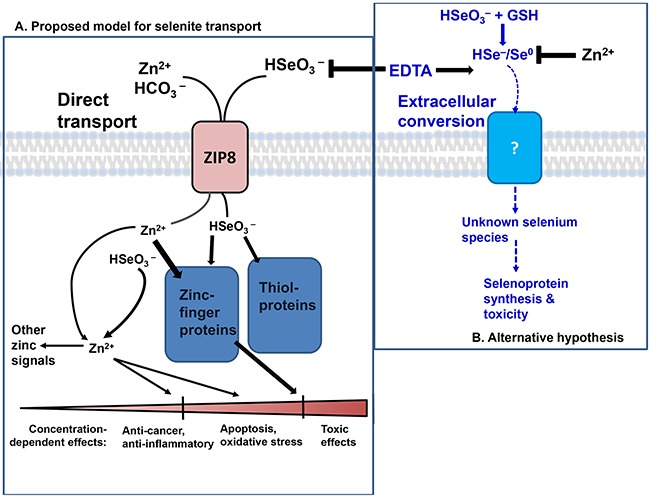
The two proposed models of selenite transport **A.** ZIP8-mediated HSeO_3_^−^ uptake model, which is both zinc- and bicarbonate-dependent. *Right*, the extracellular HSeO_3_^−^ conversion model. In the former, ZIP8-mediated HSeO_3_^−^ uptake can be prevented with prior chelation of Zn^2+^ by EDTA. Intracellular HSeO_3_^−^ reacts spontaneously with proteins containing coordinated thiol groups−−modifying signaling pathways dependent on zinc-finger proteins, or thiol proteins. Functional downstream effects on multiple targets are dependent on HSeO_3_^−^ concentration and ZIP8 levels. HSeO_3_^−^ reactions with zinc-finger proteins might release free Zn^2+^, which, in addition to Zn^2+^ transported by ZIP8, is proposed to elevate labile Zn^2+^ concentrations and participate in subsequent zinc-signaling functions. Concentration-dependent effects (*bottom*) range from anti-cancer and anti-inflammatory, to apoptosis and oxidative stress, to cytotoxicity. **B.** In the alternative model, HSeO_3_^−^ uptake is proposed to be regulated by extracellular thiol reduction of selenite–in the extracellular milieu–followed by uptake of unknown reduced Se product(s) [22, 35].

Figure 7A illustrates our proposed model of ZIP8-mediated HSeO_3_^−^ uptake, following which intracellular Se (in undetermined ionic forms) interacts with target proteins within the cell and then elicits concentration-dependent downstream effects. A particular Se level can be efficacious (anti-cancer and anti-inflammatory), higher Se levels affect the apoptosis pathway, programmed cell death, and reactive oxidative species (ROS) formation. And, sufficiently high Se levels can lead to overt toxicity and necrotic cell death [34].

Figure 7B illustrates the previously proposed extracellular HSeO_3_^−^ conversion model [22, 35]. Extracellular thiol-mediated reduction of HSeO_3_^−^ occurs first, followed by uptake of one or more unknown Se products(s). The data herein strongly support ZIP8- mediated HSeO_3_^−^ active transport (Figures 1–5) and not the extracellular thiol-mediated model (Figure 6). Figure 5A, in particular, shows that EDTA pretreatment of RBCs blocks selenite-treated cells from increased Se content; this finding supports the ZIP8-mediated selenite uptake model, and unambiguously does not support the thiol-reduction model in which EDTA shows no such inhibition, and, if anything, slight stimulation of HSeO_3_^−^ influx.

If HSeO_3_^−^ uptake is ZIP8-dependent and requires Zn^2+^ and HCO_3_^−^, how does this fact complement what is already known about the complex that ZIP8 transports into the cell? Electrogenicity experiments have shown unequivocally that ZIP8 moves Zn^2+^/(HCO_3_^−^)_2_ and Cd^2+^/(HCO_3_^−^)_2_ into the cell as an electroneutral complex [30]. If HSeO_3_^−^ is part of the zinc- and bicarbonate-dependent transport complex, the only possible electroneutral complex that can explain data in the present study would be Zn^2+^/(HCO_3_^−^)(HSeO_3_^−^).

This hypothesis will be tested in the future, with mammalian cell cultures or *Xenopus* oocytes *ex vivo*. By carefully controlling the pH as the concentration of added HCO_3_^−^ is increased, one can follow the rate of Zn^2+^ uptake; if the uptake is not linear as a function of HCO_3_^−^, then this would be strong evidence for the existence of two bicarbonate-binding sites on the ZIP8 protein. The approximate Km values of transport for the two bicarbonate-binding sites could then be estimated.

The Km value ot ZIP8-mediated transport for divalent cations (Zn^2+^, Mn^2^, Fe^2+^, Co^2+^, Cd^2+^) are known to range between 0.3 μM and 0.7 μM [27–31], yet the Km value for the HSeO_3_^−^ anion was estimated herein to be 5.9 μM. However, the two bicarbonate-binding sites on ZIP8 are likely to have very different (10-fold; or even more) Km values. There are innumerable examples in the literature of “high-affinity” and “low-affinity” binding sites for the same moiety on the same protein. Therefore, to have Km values of transport for a divalent metal cation 10-fold (or more) lower than that of HCO_3_^−^ is entirely feasible. Moreover, it is likely that HSeO_3_^−^ would displace HCO_3_^−^ at the higher Km, not the lower, bicarbonate-binding site because HCO_3_^−^ is most likely the critical driving force behind ZIP8-mediated influx.

Further questions to study might also include: can Mn^2+^, Fe^2+^, Co^2+^ and/or Cd^2+^ replace Zn^2+^ as the divalent cation for ZIP8-mediated HSeO_3_^−^ uptake? We postulate that the answer would be yes.

The present study is consistent with ZIP8 capable of moving three ions [Zn^2+^/(HCO_3_^−^)(HSeO_3_^−^)] across a membrane. Membrane-bound transporters moving three distinct ions across the cell membrane are not without precedent. The first example was NBC1 (sodium-bicarbonate cotransporter-1) encoded by the *SLC4A4* gene, which functions in the renal proximal tubule cell in the 1:1:1 cotransport of (CO_3_)**^2−^**, HCO_3_^−^, and Na^+^ on distinct binding sites [36]. Others include NKCC1 and NKCC2, encoded by the *SLC12A2* and *SLC12A1* genes, respectively. These two proteins function as cotransporters [37] for Na^+^, K^+^, and Cl^−^. NKCC1 is located in many organs that secrete fluids, whereas NKCC2 is found specifically in kidney and functions to balance the Na^+^, K^+^ and Cl^−^ ions between urine and blood. The other membrane-bound transporter known to move three distinct ions across the cell membrane is GAT1 (gamma-aminobutyric acid transporter-1) encoded by the *SLC6A1* gene [solute carrier family 6 (neurotransmitter transporter), member 1]. The ions transported by SLC6A1 [38] in nerve cells include two Na^+^, one Cl^−^ and one gamma-aminobutyric acid (GABA), removing GABA from the synaptic cleft [38].

The role of ZIP8 has been shown to be pivotal in combating lung injury and infection [39, 40]. Whereas ZIP8 over-expression in mouse cartilage tissue causes osteoarthritis—another form of inflammatory disease—osteoarthritis is suppressed in chondrocyte-specific conditional *Slc39a8(−/−)* knockout mice, with concomitant decreases in Zn^2+^ influx and matrix-degrading enzymes in chondrocytes [32].

In genome-wide association studies, a *SLC39A8* gene variant is associated clinically with low HDL-cholesterol, elevated blood pressure, increased body mass index, and abnormal natriuric peptide levels [41]. *SLC39A8* mutations have also been associated with schizophrenia [42, 43], mental retardation [44], and with cerebellar atrophy [45] or cranial asymmetry [46] with accompanying dysmorphologies. Our reason for choosing those particular mouse tissues for HSeO_3_^−^ uptake studies (Figure 4) reflected this growing list of ZIP8-related clinical disorders.

## MATERIALS AND METHODS

### Chemicals

Sodium selenite (HSeO_3_^−^), zinc chloride, reduced glutathione (GSH), ethylene-diamine-tetraacetic acid (EDTA), *N, N, N′, N′*-tetra-kis(2-pyridylmethyl)ethane-1,2-diamine (TPEN) and sodium bicarbonate (HCO_3_^−^) were purchased from Sigma. Due to its instability, HSeO_3_^−^ solutions were prepared daily in 100×-1000× stock solutions, then kept at 4°C until use. Because of its instability, HCO_3_^−^ was added immediately preceding each experiment.

### Cell cultures

Primary cell lines were prepared as described previously [32]. Briefly, from *Slc39a8(+/neo)* ×*Slc39a8(+/neo)* intercrosses, *Slc39a8(neo/neo)* embryos were isolated at gestational day 11.5, and bodies (with head, extremities, and liver removed) were suspended in phosphate-buffered saline (0.9% NaCl) (PBS) and disrupted by manual pipetting, resuspended in 2× trypsin (Sigma) for 20 min with occasional shaking, and resuspended in RPMI 1640 with 10% FBS (fetal bovine serum) and plated in 6-well plates (considered “Passage 0”, primary cultures). Mouse embryo fibroblasts (MEFs) at Passage 2 were used in assay cultures of RPMI 1640 with 10% FBS, until the transport assay—at which time RPMI was replaced with MEM-EBSS containing 25 mM HCO_3_^−^ and 1× non-essential amino acids (HyClone) medium having no supplements.

MEFs used for stable transfection were immortalized and transfected with ZIP8 cDNA (ZIP8-MEFs) or the firefly *Luciferase* gene (control LUC-MEFs) in pRevTre vectors. MEFs, cultured in MEM-EBSS medium (25 mM bicarbonate) supplemented with 10% FBS and 100 U/mL penicillin and 0.1 mg/mL, were grown in presence of puromycin for selection of the pRevTre resistance marker [28]. Prior to transport assay or expression analysis, MEFs were subcultured in medium containing 10% or 2% FBS and grown to semiconfluency.

Human prostate cancer DU145 cells (ATCC HTB-81) were cultured in RPMI-1640 (HyClone) with 10% FBS and penicillin (100 U/mL) and streptomycin (0.1 mg/mL) supplements. All other prostate cancer cell lines were obtained from either ATCC or other sources (LNCaP (ATCC CRL-1740), PC3 (ATCC CRL-1435), WPMY-1 (ATCC CRL-2854), RWPE-1 (ATCC CRL-11609); the remainder of the cell lines were cell lysates: BPH-1, C4-2B, RWPE-W99, CF-91, MLC8891 were gift from Hsueh-Liang Fu).

### Selenite-induced toxicity

Cell viability as a function of HSeO_3_^−^ concentration, was determinmed in 24-well plates with three replicates. Cells were seeded in wells, along with indicated concentrations of HSeO_3_^−^ and Zn^2+^. Viability was measured by trypan blue exclusion after 24-h incubation. Assays were performed in fresh MEM medium containing 25 mM HCO_3_^−^, and cultured at 37°C and 5% CO_2_.

### Short hairpin RNA (shRNA) knockdown of ZIP8

TAs is an established cell line, derived from chronic arsenic treatment of human bronchial epithelial BEAS-2B cells, as described (Chang *et al.*, 2014); TAs cultures were maintained in DMEM supplemented with 2% FBS, penicillin (100 U/mL) and streptomycin (0.1 mg/mL). TAs cells were transfected with lentivirus expressing specific shRNA sequences (Genecopoeia; Catalog #HSH016797-LvmU6) specifically targeted against ZIP8 mRNA; stable lines were selected by continuous culturing in 5 μg/mL puromycin. Cells were cultured and collected for Western blots; transport assays were performed using same cell cultures with sodium selenite and zinc chloride added at 200 μM for 15 min, and then quantified by inductively-coupled plasma mass-spectrometry (ICP-MS) (as described below).

### Oocyte studies *ex vivo*

*Xenopus laevis* oocytes were defolliculated with collagenase A (Roche Applied Science), and mature stage VI oocytes were isolated for microinjection. Mouse ZIP8 cDNA was cloned into pGHJ [47] for oocyte expression, which was linearized and used to synthesize capped RNA (cRNA) with the mMessage in-vitro transcription kit (Ambion); the cRNA was then microinjected into oocytes as described [48]. Oocytes were incubated at 16°C in ND96 complete buffer for 3-4 days until the transport assay. ND96 buffer contains 5 mM Hepes, 1 mM MgCl_2_, 1.8 mM CaCl_2_, 96 mM NaCl, and 2 mM KCl; a careful balance of this cation/anion ratio was always maintained (*i.e.* when adding the HCO_3_^−^ anion, Cl^−^ anion was removed to maintain equal molality). Oocytes were transferred to well plates containing ND96 buffer without magnesium or calcium and containing freshly-added 3.5 mM HCO_3_^−^ for transport assays. HSeO_3_^−^ and/or Zn^2+^ was added for 30 min and then washed in ice-cold ND96 buffer; individual oocytes were then digested in nitric acid for Se quantification by ICP-MS.

### Transport assays

For transport assays in *X. laevis* oocytes and MEFs, cells were washed and changed into buffer/medium without supplements and pre-equilibrated for 20 min. Oocytes were exposed to the indicated chemicals and incubated at room temperature for 30 min, then transferred to ice to inhibit further transport, and washed 3× in ice-cold ND96 buffer to remove external Se.

Medium for MEF cultures in well plates was replaced with Hank's Balanced Salt Solution (HBSS) containing 25 mM HCO_3_^−^, with pH confirmed as 7.5 ± 0.1 before use. Cells were exposed to the indicated chemicals and incubated at 37°C for the indicated times, then gently washed with ice-cold PBS. Fibroblasts were viable under all experimental conditions, as determined by pilot studies using trypan blue exclusion.

Transport assays in DU145 and TAs cells were carried out, following a similar protocol with indicated concentrations of Zn^2+^ and HSeO_3_^−^ added to the transport buffers. All transport assays contained 25 mM HCO_3_^−^, unless otherwise indicated.

For transport assays in mouse erythrocytes (red blood cells; RBCs), we isolated blood (0.5 mL) from the jugular vein; this was immediately diluted with PBS (5 mL containing 25 mM HCO_3_^−^) to prevent coagulation. Prior to the assay, blood was resuspended, and any coagulated erythrocytes removed. For assays using resuspended RBCs, the blood/PBS suspension was centrifuged for removal of plasma and buffy coat from the supernatant fraction, and resuspended (washed) 2× in PBS containing 25 mM HCO_3_^−^. Following addition of the indicated chemicals, RBCs were incubated in a 37°C water bath for 15 min (or indicated times), and then placed on ice and washed with ice-cold PBS to stop further transport activity.

### Se and Zn quantification

Following transport assays or IP injections, isolated tissue/oocytes/cultured cells were digested with 70% nitric acid until completely dissolved, and then diluted in deionized water for Se quantification by ICP-MS; Perkin Elmer, Nexion 300), as described [48].

### Animals

All mouse experiments were conducted in accordance with the National Institutes of Health standards for care and use of experimental animals and the University Cincinnati Medical Center Institutional Animal Care and Use Committee (protocol #05-08-11-01; approved 3 Sept 2011 - 3 Sept 2014 to DWN). At Oakland University, a similar protocol #12064/15064 was approved (1 July 2012 − 24 Aug 2018 to ZL.).

ZIP8 in wild-type (WT) mice is known to be expressed in virtually all cell types of the body [27]. In fact, ZIP8 has been used as an indicator of cell differentiation (self-renewal-related signaling) in mouse embryonic stem cells [49]. *BTZIP8*-3 mice [in mixed C57BL/6J (B6) ×129S6/SvEvTac genetic background] were generated by gDNA random insertion of three *Slc39a8*-containing BAC fragments (*Slc39a8* gene, plus flanking regions containing no other genes) in tandem. Compared with WT mice carrying two copies (diploid) of *Slc39a8* gene, *BTZIP8*-3 mice carry a total of five gene copies and exhibit ~2.5-fold increased ZIP8 mRNA and protein expression in every tissue examined [33]

The *Slc39a8(neo)* allele includes a *neo* mini-cassette retained in intron 3 [32], which, when homozygous, leads to a hypomorphic phenotype with substantially lower expression (~15% of wild-type) of ZIP8; these mice are maintained in a >99.8% B6 genetic background. Among all tissues examined, ZIP8 mRNA levels in *Slc39a8(+/neo)* are usually similar to those of the *Slc39a8(neo/neo)* rather than those of *Slc39a8(+/+)* WT mice, but exhibit high interindividual variability [32].

For intraperitoneal (IP) HSeO_3_^−^ treatment, fresh selenite was dissolved in 0.9% sterile saline (0.9% NaCl in water). Following sacrifice by CO_2_ asphyxiation, tissue for Se quantification was isolated and standardized, according to wet weight. Mice between 2-3 months of age, and similar numbers of males and females, were used. IP injection and tissue analysis were done with the experimenter blind to the genotypes.

### Antibody staining

Primary antibody for human and mouse ZIP8 detection (isoforms 1 and 2) and primary antibody for Na^+^/K^+^ ATPase subunit β-1 were purchased from Santa Cruz Biotechnology (BIGM103 (D-13), catalog #sc-133414; and Na^+^/K^+^ ATPase-β (H-3), catalog #sc48345, respectively). Western blotting for 2 h at room temperature was performed using standard procedures according to the equipment manufacturer guidelines (Bio-Rad), and with 1:2000 ZIP8 antibody dilution for MEFs and mouse tissues, except blood (1:3000). For immunofluorescence, MEFs were cultured on coverslips overnight to semiconfluency, fixed with 4% paraformaldehyde/PBS solution, blocked with 5% FBS/1% BSA/PBS, and incubated overnight at 4°C with 1:500 dilution of ZIP8 and 1:100 dilution of Na^+^/K^+^-ATPase subunit β-1 antibodies. Cells were washed in blocking buffer and incubated for 1 h with secondary IgG (H+L) F(ab′)_2_ Fragment (Alexa Fluor 488 conjugate) (Cell Signaling, catalog #4408) or rhodamine (TRITC)-conjugated goat anti-mouse IgG (H+L) (ProteinTech, catalog #SA00007-1), washed with PBS, and counterstained with 4′,6-diamidino-2-phenylindole (DAPI).

### Microscopy imaging

Z-stack confocal laser-scanning microscopy was used for imaging of cell and tissue samples, as described (He *et al.*, 2006). Fluorescence intensity was quantified using NIS Elements software.

### Reduction of selenite

HSeO_3_^−^ reduction was carried out in water by sequential addition of HSeO_3_^−^ first, then EDTA (if indicated), and finally GSH. Rate of HSeO_3_^−^ reduction was estimated by formation of the visible red elemental Se^0^ [50]. HSeO_3_^−^ reduction by GSH (4:1 molar ratio) was slowed in the presence of Zn^2+^ (1:1 ratio). Reduction of HSeO_3_^−^ during the transport assay with erythrocytes was performed by preincubating red blood cells with EDTA, and then adding GSH and HSeO_3_^−^ in an 8:1 molar ratio.

### Statistical analysis

Statistical significance between groups was determined by Student's t test using Excel. Each experiment had a minimum of three replicates and was repeated three or more times. Sigma Plot 10.0 was used for regression analysis (curve-fitting) and determination of K_m_ values. *P <0.05, **P <0.01.
